# Prenatal Drugs and Their Effects on the Developing Brain: Insights From Three-Dimensional Human Organoids

**DOI:** 10.3389/fnins.2022.848648

**Published:** 2022-03-25

**Authors:** Isidora N. Stankovic, Dilek Colak

**Affiliations:** ^1^Center for Neurogenetics, Feil Family Brain and Mind Research Institute, Weill Cornell Medicine, Cornell University, New York, NY, United States; ^2^Gale & Ira Drukier Institute for Children’s Health, Weill Cornell Medicine, Cornell University, New York, NY, United States

**Keywords:** organoids, metabolism, drugs, brain development, prenatal

## Abstract

Decades of research have unequivocally demonstrated that fetal exposure to both recreational and prescription drugs *in utero* negatively impacts the developing brain. More recently, the application of cutting-edge techniques in neurodevelopmental research has attempted to identify how the fetal brain responds to specific environmental stimuli. Meanwhile, human fetal brain studies still encounter ethical considerations and technical limitations in tissue collection. Human-induced pluripotent stem cell (iPSC)-derived brain organoid technology has emerged as a powerful alternative to examine fetal neurobiology. In fact, human 3D organoid tissues recapitulate cerebral development during the first trimester of pregnancy. In this review, we aim to provide a comprehensive summary of fetal brain metabolic studies related to drug abuse in animal and human models. Additionally, we will discuss the current challenges and prospects of using brain organoids for large-scale metabolomics. Incorporating cutting-edge techniques in human brain organoids may lead to uncovering novel molecular and cellular mechanisms of neurodevelopment, direct novel therapeutic approaches, and raise new exciting questions.

## Introduction

Recent data indicates that in the last year nearly 275 million people used illicit drugs and over 36 million people are suffering from drug abuse worldwide (United Nations Office on Drugs and Crime, 2021). As the number of drug users increases globally, in the United States, nearly 53 million Americans (19.4% of people over the age of 12) have consumed an illicit drug in the last year (National Center for Drug Abuse Statistics, 2022). Illicit drugs include marijuana/hashish, crack/cocaine, heroin, hallucinogens, inhalants, or illegally obtained prescription psychotherapeutics (Substance Abuse and Mental Health Services Administration, 2009). Among pregnant women, the use of non-illicit drugs such as alcohol and tobacco appeared to stabilize at ∼5.9% reporting use of both of these drugs in 1 year, 8.5% consumed alcohol, and 15.9% smoked cigarettes (Smith, 2021). Taken together, this resulted in over one million offspring that were exposed to an illicit drug, alcohol, or tobacco *in utero* (Smith, 2021). Additionally, over 40% of the women enrolled in the Infant Development, Environment, and Lifestyle (IDEAL) study reported continuous drug consumption into the third trimester, half of whom did not change their personal use during the entire pregnancy (Della Grotta et al., 2010). Illicit substance abuse has been linked to fetal growth restriction, stillbirth, preterm birth, and neonatal intensive care unit admission (Ross et al., 2015). However, identifying the specific molecular and cellular neurodevelopmental mechanisms underlying these outcomes is challenging. In addition, prenatal drug exposure studies are often confounded by multiple variables including multidrug usage, poverty, and a small number of participants. Thus, most neurodevelopmental outcomes are studied in adult/teenage participants, animal models, and 2D cell cultures. While many of the factors expressed in an adult brain or a neuronal culture are also present in progenitor cells, precise molecular mechanisms by which different illicit drugs shape the neurodevelopmental landscape of a fetal brain are extremely difficult to track with current approaches.

## Prenatal Drug Exposure Alters the Structure, Neurogenesis, and Connectivity of the Developing Brain

Historically, animal models have provided great insights into the neurodevelopmental consequences of prenatal drug exposure. Marijuana has become the most commonly abused illicit drug during pregnancy in the United States (Hurd et al., 2019). Previous studies in animal models demonstrated that exposure to marijuana during development may predispose children to psychiatric disorders by altering the endocannabinoid (eCB) system, which is key for the proper wiring of the brain (Harkany et al., 2007; Wu et al., 2011; Torii et al., 2017). Additionally, *in utero* exposure to TΔ-9-tetrahydrocannabinol (THC, the active ingredient in marijuana) can alter the general responsiveness of the mesolimbic dopamine system. Rats exposed to THC *in utero* exhibited changes in the ratio of excitatory and inhibitory inputs onto dopamine neurons (Frau et al., 2019; Hurd et al., 2019).

Meanwhile, the effects of *in utero* exposure to opioids on the developing brain largely depend on the type of opioid, dose, and timing of the exposure. Most commonly studied opioids include: morphine, methadone, buprenorphine, and oxycodone (Byrnes and Vassoler, 2018). Rat models of opioid exposure exhibited increased spine density and dendritic length during early development in response to morphine (Ricalde and Hammer, 1990) while changes in myelination and axon length were observed under buprenorphine and methadone treatment (Sanchez et al., 2008; Wu et al., 2014). Similarly, a series of studies reported that prenatal opioid exposure in rats caused significant changes in neurotransmitter content and post-synaptic activity (Robinson et al., 1996; Robinson, 2000, 2002).

Non-illicit drugs such as nicotine and alcohol are also commonly abused and have profound effects on the developing brain. Nicotine binds to nicotinic acetylcholine receptors (nAChR) acting as a neurotransmitter and powerful neuromodulator. Prenatal mice exposed to nicotine showed reductions in both cingulate cortex volume and dopamine turnover (Zhu et al., 2012). Meanwhile, in developing rats, nicotine led to deficits in dendritic branching, dendritic length, and spine density (Muhammad et al., 2012). Similarly, *Drosophila melanogaster* larvae developmentally exposed to nicotine displayed alterations in the dopaminergic system and brain size (Morris et al., 2018).

Animal model systems have also been critical in establishing the causal relationship between ethanol exposure and developmental brain changes. Perhaps surprisingly, prenatal ethanol exposure is not only affecting the offspring but bears a transgenerational neurodevelopmental impact (Abbott et al., 2018). Prenatal exposure to ethanol in mice also resulted in ectopic intracortical connectivity, decreased global DNA methylation levels (Badanich et al., 2013), and enhanced intrinsic excitability, glutamatergic synaptic signaling, neural progenitor cell survival, and proliferation (Nimitvilai et al., 2016). Rats prenatally exposed to ethanol showed reduced spine density, altered dendritic branching, and increased soma size in the medial prefrontal cortex (King et al., 1988), a brain region known to exert the highest baseline metabolic activity and implicated in the regulation of attention, habit formation, and working memory.

## The Effects of Prenatal Drugs on Brain Metabolism

In addition to structural and functional changes observed in animal models of prenatal drug exposure, a growing number of studies explored the brain metabolome. In rats, alteration in one-carbon (C1) metabolism was detected after fetal ethanol treatment (Ngai et al., 2015). C1 metabolism was specifically enhanced by increasing homocysteine and methionine concentrations in offspring plasma. C1 metabolism activity is necessary for healthy brain development during pregnancy and is supported by micronutrients such as methionine, choline, vitamin B12, betaine, and folate (Chen et al., 2020). The precise role of biochemical reactions that support the methylation of molecules involved in metabolic activity in brain development requires further investigation. This is an important point given that gene expression is deeply influenced by DNA methylation. In adult rats exposed to alcohol *in utero*, genes involved in the hypothalamus pituitary adrenal (HPA)-axis displayed increased gene or promoter methylation as compared to untreated rats (Ngai et al., 2015).

In other animal models, monkeys exposed to ethanol *in utero* exhibited elevated adrenocorticotropic hormone (ACTH) and cortisol (CORT), leading to increased neonatal irritability (Kraemer et al., 2008). It was also demonstrated that monkeys exposed to alcohol in middle-to-late gestation had lower primary serotonin levels and dopamine metabolites -5-hydroxyindoleacetic acid (5-HIAA) and homovanillic acid (HVA) (Schneider et al., 2011). These findings raise the question of whether abnormal serotonin biological pathways could be responsible for some of the psychiatric conditions associated with fetal alcohol spectrum disorder.

Prenatal exposure to nicotine has also been reported to cause alterations in developing brain metabolism (McCarthy et al., 2018). In a mouse model, chronic cigarette smoke exposure was linked to significant differences in amino acid, purine, lipid, fatty acid, and steroid metabolite levels as compared to unexposed offspring (Cruickshank-Quinn et al., 2014). Additionally, whereas 60% of the metabolite changes were reversible, 40% of metabolites remained altered (including nicotine metabolites) despite 2 months of smoking cessation. Taken together, these animal studies highlight the need to explore the role of dysregulated metabolites in human brain development during fetal drug exposure (Byrnes and Vassoler, 2018). Efforts in this research direction may lead to the identification of novel biomarkers or underappreciated pathogenic pathways.

## Human Models Are Needed for Studying Normal and Disordered Human Brain Development

Although animal models have provided important insights into our understanding of fetal neurodevelopment, amid the similarities of rodent and human brain development, significant differences exist. Humans and rodents share substantial stages of brain development including initial neocortical development, excitatory and inhibitory neuron development, and circuit formation (Robinson et al., 1996; Zhang et al., 2020). Although the developing nervous system is highly conserved, the trajectory and timing of differentiation, as well as maturation of transmitters and receptors, differs between humans and rodents. Additionally, while neurogenesis in humans occurs around 4.5 months of pregnancy (Silbereis et al., 2016), neurogenesis in mice is seen between embryonic days 9–18 (La Manno et al., 2020). As for prenatal substance exposure, the effects of maternal nicotine treatment on fetal neurogenesis was characterized and well described using mouse models (Zhang et al., 2020). However, in contrast to the human brain, the mouse brain exhibits lissencephaly, lacks the large pool of neural stem cells (NSCs) seen in humans, and mouse NSCs exhibit lower number of divisions before differentiation (Sarieva and Mayer, 2021).

A shift from animal to human models of *in utero* drug exposure has been previously attempted, however, these studies encountered several limitations. Primarily, human studies relied on imaging techniques such as MRI and fMRI (Roussotte et al., 2010; Nygaard et al., 2018), which capture gross brain changes. Additionally, due to the association of socioeconomic status and drug use, studies involving human subjects suffer from participant retention that possibly confounds results (Peterson et al., 2020). An alternative approach to understanding human brain disorders has been the use of human post-mortem tissue. However, post-mortem studies have offered limited insight into the time course of human brain development as well as how prenatal substance exposure impacts gross anatomical and molecular-cellular changes. More importantly, the role of metabolism in human brain development under specific conditions remains enigmatic. One key study demonstrated prenatal cannabis exposure decreased levels of the opioid peptide precursor preproenkephalin (PENK) in the caudal putamen (Wang et al., 2006). Intriguingly, nicotine and alcohol exposure had no effects on opioid peptide precursors (Wang et al., 2006). The main limitations human post-mortem drug studies encounter are referral, ascertainment bias (Peterson et al., 2020), and circumstances of death that often mask the molecular effects of treatment. Furthermore, in fetal development investigations, the primary method of tissue collection is from voluntary saline abortions which involve varying protocols that likely influence the metabolomic end results and highlight the need for more standardized models.

Recent advances in genomics, genetics, and imaging fields, support the importance of shifting from animal and post-mortem studies to more human-descriptive models of brain development research (Linsley et al., 2019). To do so, scientists have employed human embryonic stem cells (ESCs), human induced pluripotent stem cells (iPSCs) and brain organoids, which have proven as models that most faithfully and uniformly recapitulate human specific cerebral development and disease mechanisms. Stem cell-derived brain organoids demonstrate excellent potential to investigate development, disease, signaling pathways, drug toxicity, and the efficacy of various therapeutic agents. It is known that these organoids recapture organ microanatomy and mimic multicellular function at least to a certain extent. However, the field of metabolism in brain organoids is in its infancy, and understanding the role of brain metabolism in neurodevelopment and disease will require thorough evaluation.

## Three-Dimensional Cerebral Organoids as Models for Studying Fetal Brain Development

**FIGURE 1:**
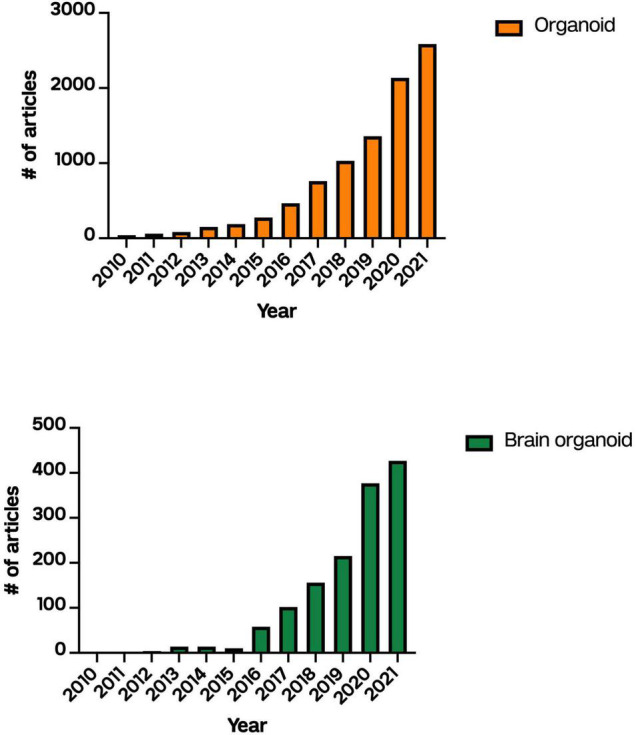
Number of articles published describing “organoids” or “brain organoids” on PubMed between 2010 and 2021.

To date, studying human brain development using fetal tissues has posed ethical considerations and experimental difficulties. One prominent technical challenge in studying fetal brain development is that fetal neocortical organotypic slice cultures can only be maintained for up to 3 weeks, thus precluding long-term interventions (Eiraku et al., 2008). Ethically, obtaining human fetal tissue for research is allowed in a very small number of cases (Sarieva and Mayer, 2021). The generation of human iPSCs from somatic cells has provided the unique opportunity to study human brain development as well as opened new avenues for precision medicine using personalized cell therapy. iPSCs retain the donor genome, may regenerate indefinitely, and undergo differentiation into virtually any cell type of interest using well-established protocols. Hence, iPSCs are an ideal system for human disease modeling. Three-dimensional (3D) culturing methods, such as the cerebral organoid technique, further enhance the utility of iPSCs to study human brain development and neurodevelopment disease (Figure 1). Using fine-tuned culturing conditions, human iPSCs spontaneously differentiate, self-assemble, and exhibit characteristics of the primitive neurepithelium at the organoid stage (Eiraku et al., 2008; Kadoshima et al., 2013).

Previous studies have shown that human iPSC-derived brain organoids mimic the developing human cerebral cortex, as they share transcriptomic, proteomic, and cellular similarities, making human brain organoids an excellent model for studying the early stages of human brain development, e.g., the first trimester of pregnancy (Lancaster et al., 2013; Lancaster and Knoblich, 2014). Of note, numerous studies have shown that 3D organoid models contain both neuronal progenitor and early-born neuron populations, as seen during early cortical development (Velasco et al., 2019; Tanaka et al., 2020), and mature neurons and astrocytes (Pasca et al., 2015; Quadrato and Arlotta, 2017). They also showcase cellular architecture observed during early corticogenesis and exhibit calcium activity (Qian et al., 2020). In general, generating 3D brain organoids relies on unguided or guided methods dictated by the scientific question of interest. Unguided differentiation of brain organoids relies on intrinsic morphogens and signaling of human iPSCs to generate brain organoids with a variety of cell lineages seen in the dorsal and ventral forebrain, midbrain, hindbrain, hippocampus, retina, choroid plexus and others (Lancaster et al., 2013; Lancaster and Knoblich, 2014; Quadrato and Arlotta, 2017). Guided differentiation utilizes small molecules and patterning factors that promote specific cell lineages to form structures resembling discrete brain regions such as cerebral cortex, cerebellum, midbrain, thalamus, hypothalamus, and others (Kadoshima et al., 2013; Yoon et al., 2019; Xu and Wen, 2021). While unguided differentiation provides opportunities to study brain region interactions, it displays high variability across organoid batches. On the other hand, guided differentiation benefits from less variation in batches and cell lines at the cost of limiting the heterogeneity observed in the self-organizing developing brain.

Regardless of the method of differentiation, recent studies have reported that spontaneous excitatory post-synaptic currents can be detected in organoid cultures starting at 4 months *in vitro* (Li et al., 2017). Moreover, organoid models are also suitable to investigate neuronal network formation at early or late stages of brain development and exhibit spontaneous network oscillations similar to those observed in pre-term human electroencephalography (Trujillo et al., 2019).

## Evaluating the Proteomic and Metabolomic Effects of Prenatal Drugs on Offspring Using Three-Dimensional Organoids

In the last 2 years, brain organoids have revolutionized how we study the developmental effects of prenatal drug exposure. To date, several reports (Wang et al., 2018; Ao et al., 2020; Dang et al., 2021; Notaras et al., 2021) have been published establishing human brain organoids as a valuable model system to identify the impact of prenatal drugs on human brain development.

Notaras et al. (2021) aimed to determine prenatal signatures that arise from narcotic use and environmental risk factors in human iPSC-derived forebrain organoids. Using this human-derived model, they studied exposure to opiates (μ-opioid agonist endomorphin), cannabinoids (WIN 55,212-2), alcohol (ethanol), smoking (nicotine), chronic stress (human cortisol), and maternal immune activation (human IL17a). Of the mimetics tested, the cannabinoid agonist WIN 55,212-2 was shown to significantly impact normal cortical development by inducing DNA fragmentation and increasing cell death in organoids. At a metabolic level, all treatment groups showed differential expression of L-phenylalanine. This is of note, as previous studies in rats have shown that phenylalanine can induce neuronal death (Li et al., 2010; Zhu et al., 2017) as well as contribute to oxidative stress in the developing brain (Fernandes et al., 2010). Additionally, organoids that were treated with ethanol, endomorphin, and nicotine exhibited differential expression of GTP. Hydrolysis of GTP into 7,8-DHNP-3′-TP is required for the biosynthesis of numerous monoamine neurotransmitters (Notaras et al., 2021) and GTPase activators are ubiquitously expressed during corticogenesis. One can postulate that differential expression of GTP upon drug enviromimetic treatment is likely to hold important implications for cortical development and synaptic plasticity later in life. Taken together, this study is pivotal for both uncovering novel cellular mechanisms of prenatal drug action and establishing 3D brain organoids as a system for evaluating the effects of illicit and non-illicit drugs on the developing brain.

Two other studies linked novel pathways to methamphetamine (METH) and ethanol prenatal exposure. Dang et al. (2021) combined 10-month-old ESC-derived organoids with single-cell RNA sequencing technologies to evaluate the effects of prenatal METH exposure on the developing brain. A robust transcriptional response within astroglial cell types in response to METH treatment was observed. Metabolically, METH treatment disrupted cAMP signaling and glutamate regulation in organoids. Intriguingly, enhanced IL-8, IL-6, GFAP, and NLRP levels were detected that ultimately elicited gliosis, neuroinflammation, and neurotoxicity in cerebral organoids (Dang et al., 2021). To our knowledge, this study was the first report describing how METH treatment elicits neuroinflammation in a human-derived model of the developing brain. Similarly, Zhu et al. (2017) modeled prenatal ethanol exposure (PAE) *in vitro* using brain organoids. Zhu et al. observed that upon ethanol exposure brain organoids displayed attenuated neurite outgrowth, disrupted neural maturation, and altered gene expression involved in organogenesis, synaptic plasticity, neural transmission, stem cell proliferation, and differentiation. This study was also the first to link GSX2, RSPO2, and the Hippo signaling pathway to ethanol-induced impaired neurogenesis (Zhu et al., 2017).

Another novel approach is combining brain organoid techniques with microfluidic device technology to evaluate the effects of prenatal drugs on the developing brain. Wang et al. (2018) took this approach to study the effects of nicotine exposure on brain development, while Ao et al. (2020) perfused brain organoids with THC to evaluate the impact of cannabinoids on corticogenesis. In a brain organoid model of nicotine exposure, another group observed abnormal neuronal differentiation and migration suggesting nicotine elicits impaired neurogenesis during early fetal brain development in humans. Meanwhile, in the presence of THC, cerebral organoids exhibited reduced neuronal maturation, downregulation of cannabinoid receptor type 1 (CB1) receptors, impaired neurite outgrowth, and decreased spontaneous firing (Wang et al., 2018).

**FIGURE 2:**
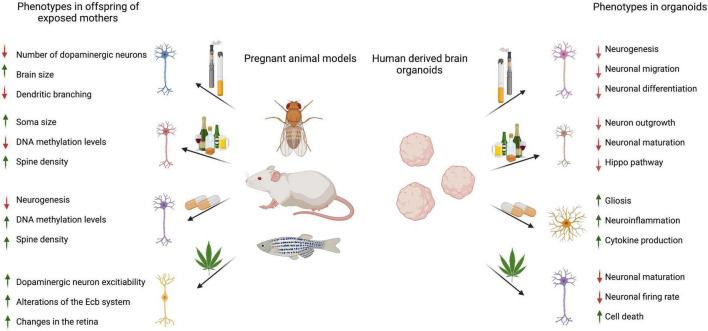
Summary of referenced animal and brain organoid studies describing the effects of prenatal drugs on offspring brains or 3D brain organoids. Image created using BioRender.com.

Taken together, these studies pioneered the approach of using brain organoids for screening commonly abused drugs during pregnancy. While the cellular mechanisms, signaling pathways, and metabolites identified in developing brain models are toxin-specific, drug screens show an effect on neurons, astrocytes, and other glial cells. Neurons often displayed impaired neurogenesis, maturation, and migration, while studies evaluating other cell types described cellular toxicity and inflammation (Figure 2).

## Future Prospects and Challenges of the Brain Organoid Field

Three-dimensional brain organoids are slowly emerging as the leading model for studying human neurodevelopment and hold promise in advancing our understanding of fetal neurobiology in health and disease. Due to their small size and short generation time, researchers can produce hundreds to thousands of organoids from relatively small batches of stem cells. Using human-derived tissue allows for rapid drug screening of candidate therapeutics that may have been incorrectly discarded in animal trials. Importantly, the broader application of organoid technology outside of the academic setting has several challenges and limitations that will need to be resolved (Chen et al., 2019). One limitation is the batch-to-batch variability of cerebral organoids, despite derivation from cells of the same donor. Methods to enhance cellular diversity in organoids will also need to be developed as the technology advances. In this regard, groups have managed to grow organoids for long periods of time to promote the generation of various cell types (Stratoulias et al., 2019; Sun et al., 2021). However, certain brain-specific cell types such as microglia remain challenging to populate in cerebral organoids (Stratoulias et al., 2019). Brain organoids also cannot fully reproduce the spatial organization of neurons observed in the human brain. Nevertheless, neurons in organoids display various organizational structures that resemble the cortical plate (Lancaster et al., 2013; Lancaster and Knoblich, 2014). In addition, recapitulating the interaction of neurons and the blood-brain barrier in the organoid system requires further exploration. Thus, we can postulate that brain organoids are better suited to model specific neurodevelopmental diseases and facilitate the testing of compounds that do not depend on distinct cortical plate organization.

As novel technologies continue to emerge, interdisciplinary approaches that combine brain organoids with microfluidic chip devices are in sight. Such organoid-on-a-chip model has provided a promising platform to interrogate neurodevelopmental disorders under environmental exposure (Wang et al., 2018; Ao et al., 2020). Moreover, the organ-on-a-chip strategy offers advantages including relatively low cost, precise control of the microenvironment (i.e., external stimuli and mechanical fluidic cues), and tissue longevity. Successful applications of organ-on-a-chip models have been highlighted in liver, lung, and hear tissues (Huh et al., 2010; Ribas et al., 2016; Moradi et al., 2020). Meanwhile, the extension of organ-on-a-chip in neurodevelopmental disease and drug screening is on the horizon. Additionally, with the explosion of omics techniques, solving a decade-long question on the exact molecular alterations caused by drug exposure during fetal brain development is becoming attainable. 3D brain organoids represent the most faithful and ethical model of human development to date, and combining this approach with omics techniques will only begin to scrape the surface of human brain development in health and disease. In this regard, evaluating how distinct metabolites regulate gene expression programs to ultimately influence trajectories of the developing brain is within our grasp.

## Author Contributions

IS and DC came up with the topic of the review and wrote the manuscript. Both authors contributed to the article and approved the submitted version.

## Conflict of Interest

The authors declare that the research was conducted in the absence of any commercial or financial relationships that could be construed as a potential conflict of interest.

## Publisher’s Note

All claims expressed in this article are solely those of the authors and do not necessarily represent those of their affiliated organizations, or those of the publisher, the editors and the reviewers. Any product that may be evaluated in this article, or claim that may be made by its manufacturer, is not guaranteed or endorsed by the publisher.
